# Evaluation of diverse soybean genotypes for seed longevity and its association with seed coat colour

**DOI:** 10.1038/s41598-023-31071-3

**Published:** 2023-03-15

**Authors:** Naflath T. V., Rajendraprasad S., Ravikumar R. L.

**Affiliations:** 1 Department of Seed Science and Technology, College of Agriculture, UAS, GKVK, Bangalore, Karnataka 560 065 India; 2 Department of Plant Biotechnology, College of Agriculture, UAS, GKVK, Bangalore, Karnataka 560 065 India

**Keywords:** Genetics, Plant sciences

## Abstract

Sixty genotypes with different seed coat colour and seed sizes were evaluated for seed longevity under both natural and accelerated ageing over seasons. The genotypes were grown during rabi, 2018, and summer, 2021, and freshly harvested seeds were used. For natural ageing, seeds were stored in a cloth bag in ambient condition and were removed at bimonthly intervals till 20 months. Accelerated ageing was carried out as per International Seed Testing Association (ISTA) guidelines. The germination percentage after natural and accelerated ageing over two seasons was determined. The correlation between two seasons of accelerated ageing and month-wise natural ageing was highly significant. The principal component analysis (PCA) using seed longevity grouped the majority of black genotypes into a separate cluster. Higher seed longevity was associated with black seed coat colour and small seed size. Microsatellite marker-based clustering also produced a separate cluster for majority of black genotypes and grouped the genotypes into a large number of clusters suggesting high diversity in the plant material. Two black seed coat colour genotypes, ACC No.369 and ACC No.39 consistently showed higher longevity under natural and both the years of accelerated ageing and serve as a source of alleles for higher seed longevity in soybean.

## Introduction

Soybean (*Glycine max* (L.) Merrill) is one of the major oilseed crops belonging to the family Fabaceae, subfamily Papilionoidea, and genus *Glycine.* The crop originated in Southeast Asia and was primarily domesticated by China around 1100 BC. Later it was introduced to several countries including India in the first century AD^1^. Soybean seeds are composed of 20% oil and 40% protein and contribute 70% to world’s protein and 28% to world’s oil consumption^2^. It is grown in an area of 119.0 million hectares with a production of 339.0 million metric tonnes worldwide and India produces 9.3 million metric tonnes from an area of 11.64 million hectares^3^. Due to its importance as a staple source of protein and oil for the growing population of the world, resources were allocated in the breeding effort to improve the grain yield with wide adaptability. A large number of varieties with high yield, early maturity, wide adaptability, and suitable for diverse agro-climatic conditions are developed in India and elsewhere^4^. Soybean seeds are very sensitive to production^5,6^, storage conditions^7,8^, seed handling, and mechanical damage^9–11^. The seed embryo and embryonic axis are located under a very thin seed coat and the surface of seed coat has presence of pores^12,13^. Hence, the seed viability and longevity are low^14,15^.

Soybean seed longevity is a matter of high concern for farmers, traders, and seed producers^14^. Seed viability is a serious concern worldwide^7,16,17^. Soybean seeds deteriorate faster and lose viability during storage^18–20^. Lipid auto-oxidation and subsequent increase of free fatty acid content are the most mentioned reasons for ageing damage of soybean seeds^21,22^. Remarkable genotypic diversity for a decline in seed longevity during storage has been well documented in soybean^23–26^. Seed longevity is a polygenic trait and the identification of genotypes with high seed longevity is very important. Earlier studies indicated that soybean genotypes with dark and hard seed coat^27–29^, smaller seed size^30^, lesser space between seed coat and cotyledons^13^, and wild type accessions^17,23^ possess higher seed longevity as compared to other seed coat colour genotypes. Such soybean genetic resources provide a valuable source for the identification of genotypes suitable for seed longevity improvement in soybean. It will broaden the genetic base for breeding programmes to combine seed longevity with seed yield per plant.

An efficient screening technique to identify the genotypes with higher longevity in a short time to select the lines in breeding is imperative in crop improvement programmes. Natural ageing not only takes longer time^17,24,26^ but also influenced by storage conditions^31,32^. Alternatively accelerated ageing technique has been proposed for testing seed germination in general^33–35^ and soybean in particular^24,28,36–38^. The protocol for accelerated ageing has been standardized for soybean^39^ which may be employed for predicting the viability of soybean seeds.

Genetic diversity information in the source germplasm is important to develop new cultivars with seed longevity. Simple sequence repeats (SSR) markers which are ubiquitously distributed within genomes are used to identify genetic diversity between genotypes in a germplasm collection^40–42^. The SSR markers have been applied in various aspects of molecular research such as genetic diversity assessment, fingerprinting, marker assisted selection, gene flow, and genetic linkage mapping^43^.

The identification and development of high seed longevity genotypes in soybean is a requirement to be used as a source of germplasm for seed longevity in breeding. With this background, the present investigation was undertaken to elucidate the information on genetic and molecular diversity among the selected germplasm and identify genotypes with higher seed longevity through accelerated and natural ageing techniques and their association with seed coat colour and seed size.

## Materials and methods

### Plant material

Sixty diverse genotypes including 46 yellow, three green (104-31, JS 90-41, and BNS-5), and nine black seed coat colour (Pune 14, Pune 30, Kalitur, ACC Nos. 37, 369, 39, 101, 109, and LB-5) were collected from author affiliated government institute, viz., All India Co-ordinated Research Project (AICRP)-Soybean scheme, Zonal Agricultural Research Station, GKVK, Bangalore, for the study and no permission is required for the collection. The genotypes, their name, and seed characteristics are given in Supplementary Table S1 and can be obtained by contacting the first author. The genotypes also differed for seed size, growth habit, and other quantitative traits related to crop growth and seed yield. The genotypes were grown and multiplied before the start of the experiment at AICRP-National Seed Project, University of Agricultural Sciences, Bangalore, during *Rabi*, 2018, and in *Summer*, 2021. Harvesting was done during January, 2019 and April, 2021, respectively for *Rabi*, 2018 and *Summer*, 2021. The seeds were immediately dried to a safe moisture content of 9% after manual threshing and the germination percent of the seeds were recorded before the start of the seed storage experiment.

### Seed longevity

The seeds obtained from *Rabi*, 2018 experiment were tested for seed longevity under both natural and accelerated ageing, while the seeds harvested in *Summer*, 2021 were tested for longevity using accelerated ageing at AICRP-National Seed Project, University of Agricultural Sciences, Bangalore.

#### Natural ageing

Freshly harvested seeds were dried uniformly to 9% moisture content and stored in a cloth bag from February, 2019 to September, 2020 under ambient conditions (25–30 °C and 60–65% RH). At bimonthly intervals from the 8th month onwards till the 20th month after harvest, the seed samples were drawn from the stored cloth bags and used for testing the germination. The germination was recorded till the 20th month after harvest. The laboratory germination test was carried out as per the International Seed Testing Association (ISTA) guidelines^44^ using between paper method. One hundred seeds in four replicates were randomly drawn from the cloth bag for each genotype at bimonthly intervals and kept for germination following between paper method. The seeds were kept in an incubator and the constant temperature of 30 °C and relative humidity of 90 ± 2% were maintained. The seeds were allowed to germinate and grow for 5 days. On the 5th day, the germination papers were removed from the incubator and the germination count was recorded following ISTA guidelines and expressed in percentage.

#### Accelerated ageing

Freshly harvested seeds after drying to 9% moisture content were used for the study in both seasons. Forty-two gram seeds for each genotype were placed in an ageing box with a wire mesh screen by following the guidelines specified by ISTA^39^ for soybean. Four varieties, DSB 32, JS 95-60, Hardee, and DSB 33, were used as checks for the experiment. The box is filled with 40 ml of distilled water and it was sealed all around to maintain more than 95% relative humidity. The box was kept in an ageing chamber which maintain a constant temperature of 41 ± 0.3 °C for 72 h. After 72 h, the treated seeds were removed from the box, weighted, and kept for germination as mentioned above in four replications immediately (within an hour). The experiment was replicated twice.

### Genetic diversity analysis

#### DNA isolation

The genotypes were grown in paper cups and the genomic DNA was isolated from young leaf tissues using cetyl trimethylammonium bromide (CTAB) method^45^. The quality and quantity of DNA were estimated using 0.8% agarose gel electrophoresis with ethidium bromide staining and the DNA was uniformity diluted to 50 ng μl^−1^ and stored.

#### Microsatellite marker genotyping

##### Primers

Fifteen microsatellite primer pairs were chosen for the study. A list of selected primers, primer sequence, and standardized annealing temperatures are given in Supplementary Table S2.

##### Genotyping

The isolated DNA was subjected to a polymerase chain reaction (PCR) using a reaction mixture of 10 μl and 38 reaction cycles. Three percent agarose gel with 0.5 μg ml^−1^ ethidium bromide was used to visualize the marker alleles along with a 1000 bp ladder to score the fragment size.

### Statistical analysis

#### Seed longevity

The germination percentage values were transformed using arcsine transformation to stabilize the variance. The analysis of variance (ANOVA) for the observed germination values for all the months and accelerated ageing was done by following a completely randomized design using SPSS software. PAST 4.03 software was used for plotting box plots and principal component analysis (PCA). Box plot was developed using the range, median, first, and third quartile values. PCA was undertaken based on scores of germination values from different storage months of natural ageing and accelerated ageing over two seasons. Eigen values and principal components were estimated using the germination values based on non-rotated loadings.

The correlation between two seasons accelerated ageing data and month-wise natural ageing data was determined using Pearson’s correlation coefficient. Further, the genotypes were classified into different longevity groups^46^ based on their germination values in natural and accelerated ageing as given below,

**Table:** 

Classes of genotype	Seed longevity rank (SR)
$\left(\mu +2\sigma \right) \,and\, above$	SR 1
$(\mu +\sigma )\text{ to }\left(\mu +2\sigma \right)$	SR 2
$(\mu -\sigma )\text{ to }\left(\mu +\sigma \right)$	SR 3
$(\mu -2\sigma )\text{ to }\left(\mu -\sigma \right)$	SR 4
$\left(\mu -2\sigma \right)\, and\, less$	SR 5

Here, ‘*μ*’ and ‘*σ*’ are the mean and standard deviation of the overall germination of genotypes, respectively.

The seed longevity ranks ‘SR 1’ and ‘SR 5’ reveals “highest” and “lowest” seed longevity, respectively. The genotypes which showed consistently higher ranking in both natural and accelerated ageing were considered as genotypes with higher longevity.

#### Molecular data analysis

Marker scoring was done manually as 0/1 matrix. The presence of an allele was denoted as ‘1’ and absence as ‘0’. The band size matrix was used for the microsatellite genotypic data analysis. Allele frequency, genetic diversity and Polymorphism Information Content (PIC) were estimated using PowerMarker v 3.25 software. A number of different alleles (Na), number of effective alleles (Ne), Shannon’s information index (I), observed heterozygosity (Ho), expected heterozygosity (He), unbiased expected heterozygosity (uHe), and fixation index (F) was computed in GeneAlEx v 6.5^47^. Dissimilarity index was calculated using Euclidean distance with 1000 bootstraps and with this, hierarchical clustering of genotypes was done based on the unweighted pair group method with arithmetic mean (UPGMA) in DARwin 5.0 software.

## Results

### Seed longevity

The longevity of a seed is measured using its germination ability after a period of dry storage. A reduction in germination percentage of the genotypes was observed throughout natural storage under ambient conditions. The average germination % of genotypes after 8 months of ambient storage was 82.34 and it gradually declined to 3.35% after 20 months (Table 1). By 14th month, CAT-44 and 104-31 genotypes lost their viability completely, whereas, ACC Nos.369, 39, and 101 maintained 100% germination. The germination of genotypes after 20 months of storage was ranging from 0.00 to 54.54%. The accelerated ageing also significantly reduced the germination of genotypes and the mean germination across genotypes was 48.16% in 2019, and 44.92% in 2021.

**Table 1:** Mean and range of seed germination percentage in different soybean genotypes after natural and accelerated ageing.

	Natural ageing (months)							Accelerated ageing	
	8	10	12	14	16	18	20	AA, 2019	AA, 2021
Mean	82.34 ± 3.26 (57.31)	68.01 ± 8.83 (44.55)	49.14 ± 7.59 (31.01)	39.06 ± 5.85 (25.39)	25.99 ± 4.31 (16.35)	11.15 ± 3.19 (6.67)	3.35 ± 1.34 (1.96)	48.16 ± 5.63 (29.77)	44.92 ± 7.34 (27.83)
Range	56.83 to 100 (34.63 to 90.00)	7.55 to 100 (4.33 to 90.00)	1.07 to 100 (0.62 to 90.00)	0 to 100 (0.00 to 90.00)	0 to 100 (0.00 to 90.00)	0 to 79.09 (0.00 to 52.27)	0 to 54.54 (0.00 to 33.06)	0 to 100 (0.00 to 90.00)	3 to 92.02 (1.72 to 66.96)

Values in the parenthesis are arcsine transformed germination percentage.

**Figure 1:**
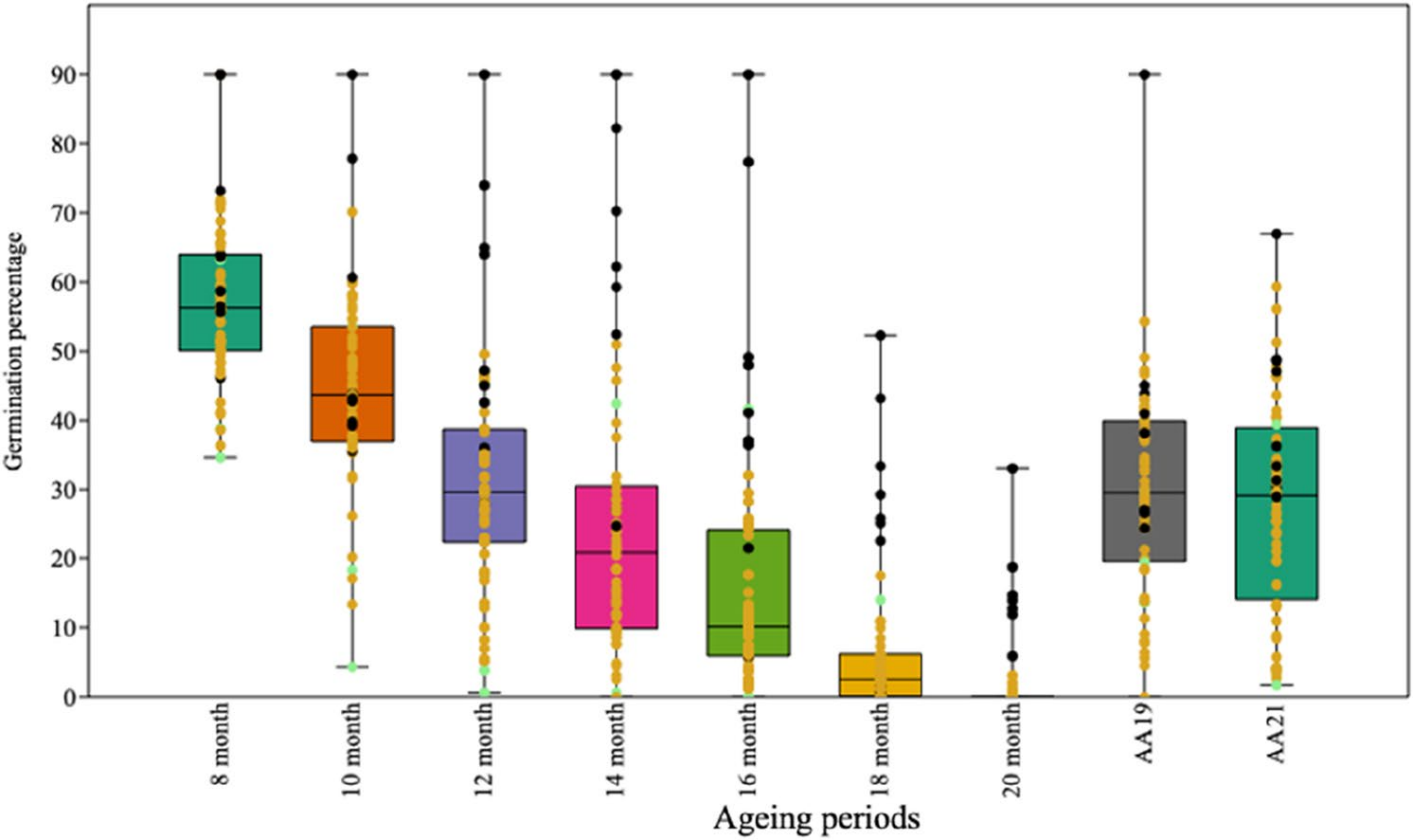
Box and jitter plot of germination percent after natural (8–20 months) and accelerated ageing (AA) (Black, Green, and Yellow colour dots indicate Black, Green, and Yellow seed coat colour genotypes).

The analysis of variance suggested highly significant variation among genotypes for germination after ageing (Table 2). The box and jitter plot drawn for germination indicated high diversity among soybean genotypes for seed longevity (Fig. 1). The genotypes in the 12th, 14th, 16th and 18th month as well as accelerated ageing in 2019 were more varied toward the positive quartile, on the other hand, in 8th and 10th month of natural ageing and accelerated ageing in 2021, the variation between the genotypes were distributed towards first and third quartiles. In all the ageing methods, the black seed coat colour genotypes (indicated as black dots in the box plot) had comparatively higher seed longevity along with a few yellow and one green colour genotypes (BNS 5), which are depicted as yellow and green colour dots, respectively.

**Table 2:** Mean sum of squares of seed germination percentage in different soybean genotypes after natural and accelerated ageing.

Source	df	Natural ageing (months)							Accelerated ageing	
		8	10	12	14	16	18	20	AA, 2019	AA, 2021
Genotypes	59	452.66**	647.62**	947.46**	1553.17**	1448.22**	358.42**	100.36**	1041.16**	780.79**
Error	120	21.51	65.13	42.99	24.60	11.74	4.13	0.63	13.93	23.76

Significance codes: **p = 0.01.

### Principal component analysis (PCA)

**Figure 2:**
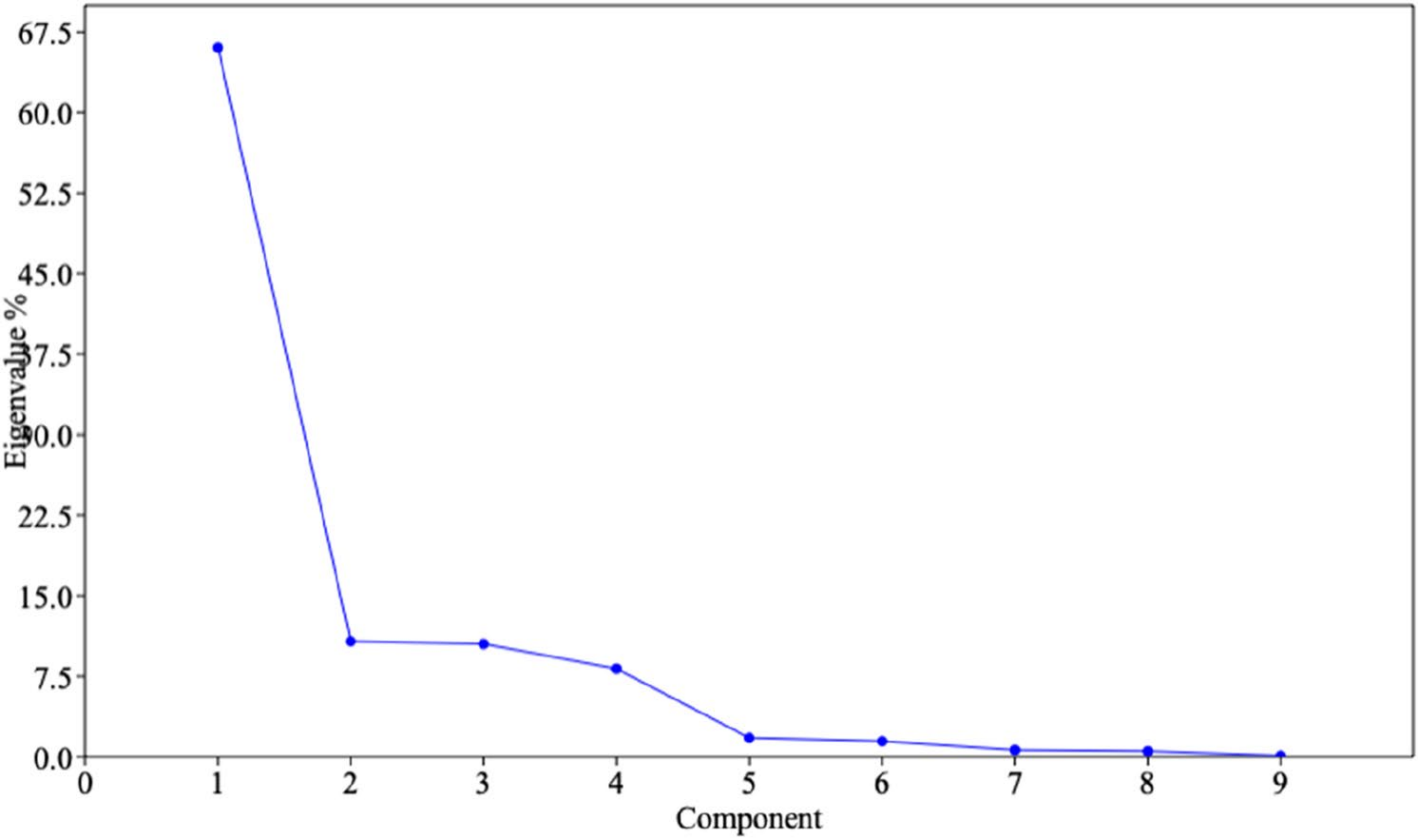
Scree plot of principal components against Eigenvalue.

**Figure 3:**
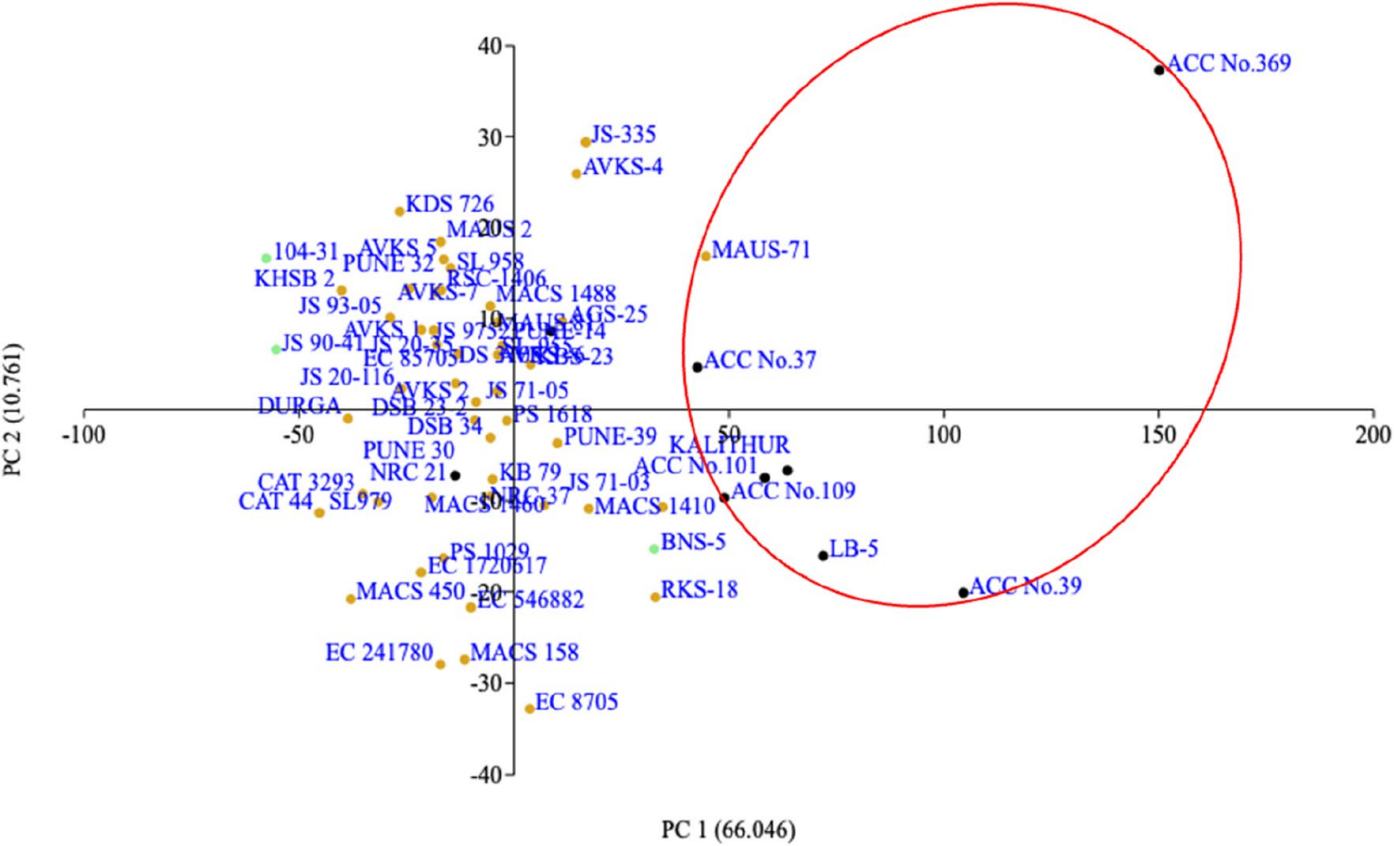
Scatter plot of soybean genotypes based on PC1 and PC2 (Black, Green, and Yellow colour dots indicate Black, Green, and Yellow seed coat colour genotypes).

PCA is an alternative method of population structure analysis and it gives the explicit pattern of combination between the genotypes in the factor plane. In the PCA analysis of 60 soybean genotypes, the eigenvalues were higher for components one and two explaining 66.046 and 10.761% variance of the population, respectively (Table 3, Fig. 2). A scatter plot was made based on the loadings of each genotype in principal components one and two (Fig. 3). Based on the similarity of the genotypes for the seed longevity, different clusters were formed in the scatter plot. ACC No. 369 genotype had a higher loading value and was found to have higher longevity. Other black genotypes such as, ACC Nos. 37, 101, 39, 109, Kalitur, and LB-5 were closely formed in a single cluster with higher scores in component 1. The genotypes with higher seed longevity are spotted in quadrants one and four, having the majority of the black seed coat colour genotypes. One green colour genotype (BNS-5) and a few yellow seed coat colour genotypes such as, MAUS-71, MACS 1410, and RKS-18 were also placed in the same quadrant along with black seeded genotypes. A wide cluster was formed in the second and third quadrant including a majority of the yellow seeded genotypes along with two green (104-31 and JS 90-41) and two black (Pune 14 and Pune 30) seed coat colour genotypes.

**Table 3:** Principal component and their respective Eigenvalue and per cent variance.

Principle component	Eigenvalue	%Variance	Cumulative % variance
1	79,854	66.046	66.05
2	13,010	10.761	76.81
3	12,714	10.516	87.32
4	9926	8.2097	95.53
5	2132.4	1.7637	97.29
6	1753.3	1.4501	98.74
7	760.59	0.62907	99.37
8	629.86	0.52095	99.89
9	126.24	0.10442	100

### Association between natural and accelerated ageing and identification of genotypes with higher longevity

**Figure 4:**
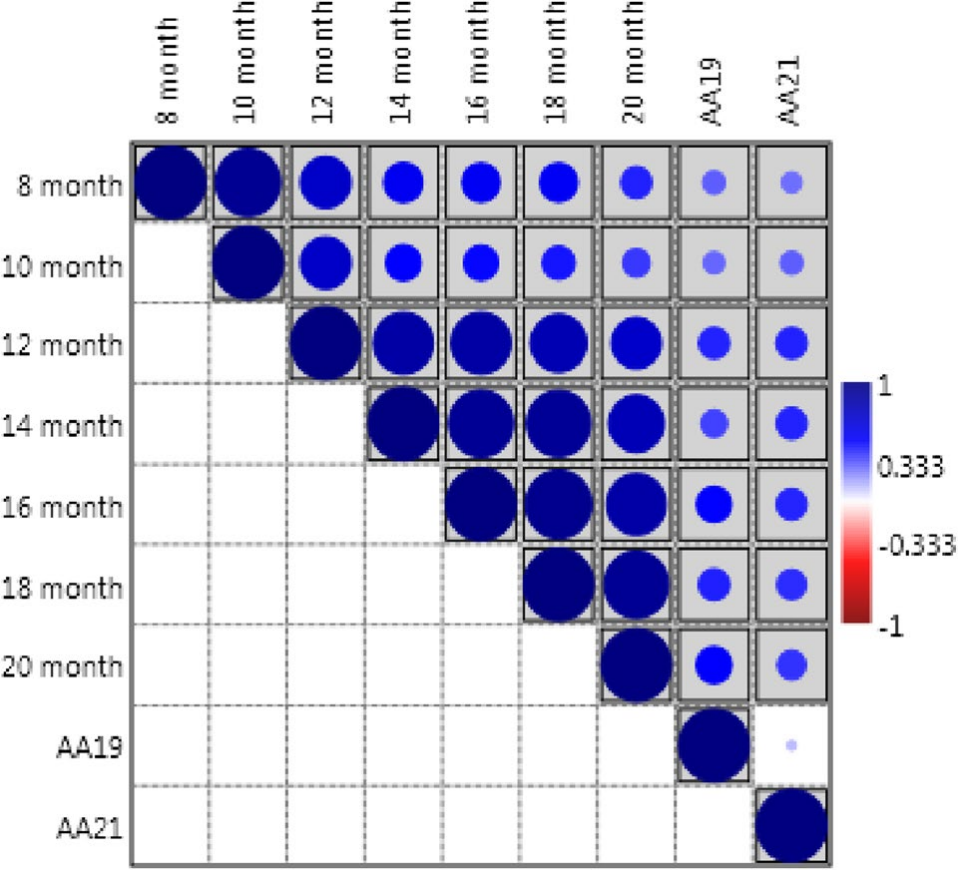
Correlation matrix of natural ageing (8–20 months) and accelerated ageing (*AA* accelerated ageing).

Pearson’s correlation was performed to study the association between natural and accelerated ageing methods to test the reliability of accelerated ageing for screening the soybean genotypes for seed longevity. A significant positive correlation was observed between germination after natural ageing with germination after accelerated ageing in both the years (Fig. 4). The correlation was significant at 8th and 10th month of natural storage, on the other hand, it was found to be highly significant after 12, 14, 16, 18, and 20 months of natural ageing with accelerated ageing in both the years. During the year 2019, the highest correlation was observed after 20th month of natural ageing (0.506) followed by 16th month of natural ageing (0.496). Similarly, during the year 2021, a higher correlation was found in 12th month (0.433) followed by 14th (0.432), and 16th (0.429) months.

The genotypes were grouped into five longevity ranks using the empirical rule which is also called as three sigma rule. The number and list of genotypes in each of the seed longevity classes are given in Table 4. The highest number of genotypes fell into SR 3 rank in both natural and accelerated ageing. More than 40 genotypes were grouped in SR 3 rank in all the months of testing under natural ageing. Forty two genotypes in accelerated ageing during the year 2019 and 38 genotypes during 2021 were observed in SR 3. None of the genotypes were found in SR 5 rank except 104-31 and KHSB 2 genotype in natural ageing after 10 months. The SR 1 rank consists of genotypes with the highest longevity. Two genotypes, ACC Nos. 39 and 369 were consistently found in SR 1 rank in most of the months of natural ageing. Under accelerated ageing also, the genotype ACC No. 369 in 2019, and the genotype ACC No. 39 in 2021 were found in SR 1 rank. Both of them are black seed coat colour genotypes. Genotypes viz*.*, ACC No. 37, 109, LB-5, MAUS 71, and RKS 18 were consistently found in SR 2 rank of seed longevity. SR 1 and SR 2 rank classes are considered better for identifying genotypes for higher seed longevity. All the black seed coat genotypes, i.e., ACC Nos. 369, 39, 37, 109, and LB-5 were consistently found under either SR 1 or SR 2 rank. It is interesting to note that the seed size of the black seed coat colour genotypes which were grouped in higher longevity was small compared to other black seeded genotypes (Pune 14 and Pune 30) which were grouped in lower seed longevity rank (SR 3) (Supplementary Table S1). None of the green seed coat colour genotypes were consistently found in higher longevity rank classes. SR 1 rank has only consisted of black seed coat colour genotypes and none of the yellow colour genotypes were found in that ground except MAUS 71 in 8th month of natural storage. Even in SR 2 class, no single yellow seed coat colour genotypes was found constant in all the natural ageing months and accelerated ageing method of testing for seed longevity.

**Table 4:** Ranking of genotypes in to different seed longevity classes in soybean based on natural and accelerated ageing.

Seed longevity class	Natural ageing (months)							Accelerated ageing	
	8	10	12	14	16	18	20	AA, 2019	AA, 2021
Seed longevity Rank-1	ACC No. 369 LB-5 MAUS-71 (3)	LB-5 ACC No. 369 (2)	ACC No.39 ACC No. 369 KALITUR (3)	ACC No. 369 ACC No.101 ACC No. 109 ACC No.39 (4)	ACC No.39 ACC No. 369 (2)	ACC No.39 ACC No. 369 ACC No.101 LB-5 (4)	ACC No.39 ACC No. 369 ACC No.37 LB-5 (4)	ACC No. 369 (1)	ACC No.39 (1)
Seed longevity Rank-2	MACS 1488 MACS 1460 DSB 34 PS 1029 KALITHUR (5)	RKS-18 DSB 34 KALITHUR MAUS-71 (4)	LB-5 MACS 1460 (2)	MAUS-71 ACC No. 37 KALITHUR LB-5 RKS-18 (5)	ACC No.37 LB-5 ACC No. 109 KALITHUR ACC No. 101 BNS-5 (6)	ACC No. 109 ACC No. 37 KALITHUR (3)	ACC No. 109 ACC No. 101 (2)	ACC No. 37 MAUS-71 PS 1618 JS-335 AVKS-4 (5)	EC 241780 RKS-18 ACC No. 109 PS 1618 LB-5 ACC No. 369 EC 8705 MACS 1410 EC 1720617 (9)
Seed longevity Rank- 3	BNS-5 AVKS-4 MACS 1410 RKS-18 ACC No. 101 ACC No.39 PUNE 30 AVKS 2 EC 546882 DS 3105 MAUS 81 DSB 23-2 AVKS -6 PUNE-14 EC 8705 AVKS-7 MACS 158 JS-335 AGS-25 KBS-23 PUNE-39 NRC-37 JS 9752 JS 71-05 JS 71-03 EC 85705 MAUS 2 AVKS 5 PS 1618 SL 958 RSC-1406 AVKS 1 KB 79 ACC No. 37 ACC No. 109 SL-955 MAUS 2 JS 93-05 JS 20-116 EC 241780 SL979 NRC 21 EC 1720617 MACS 450 JS 20-35 PUNE 32 (45)	ACC No.39 ACC No.109 ACC No.101 BNS-5 JS 71-03 ACC No. 37 PUNE 32 EC-241780 SL979 NRC 21 AVKS 2 EC 546882 DS 3105 PS 1029 JS 93-05 MAUS 81 DSB 23-2 AVKS -6 PUNE-14 EC 8705 AVKS-7 MACS 158 JS-335 JS 20-35 AGS-25 KBS-23 SL-955 PUNE-39 NRC-37 JS 9752 JS 71-05 EC 85705 PUNE 30 MAUS 2 AVKS-4 EC 1720617 MACS 1488 AVKS 5 MACS 1460 PS 1618 JS 20-116 KDS 726 SL 958 RSC-1406 AVKS 1 MACS 1410 KB 79 MACS 450 (48)	ACC No.101 BNS-5 MAUS-71 ACC No. 37 ACC No. 109 EC-241780 SL979 NRC 21 AVKS 2 EC 546882 DS 3105 PS 1029 JS 93-05 MAUS 81 DSB 23-2 AVKS -6 PUNE-14 EC 8705 DSB 34 AVKS-7 CAT 3293 MACS 158 RKS-18 JS-335 AGS-25 KBS-23 SL-955 PUNE-39 NRC-37 JS 9752 JS 71-05 JS 71-03 EC 85705 PUNE 30 MAUS 2 AVKS-4 MACS 1488 AVKS 5 PS 1618 JS 20-116 KDS 726 SL 958 RSC-1406 AVKS 1 MACS 1410 KB 79 (46)	EC-241780 SL979 NRC 21 PUNE 32 AVKS 2 EC 546882 DS 3105 PS 1029 JS 93-05 MAUS 81 DSB 23-2 AVKS -6 PUNE-14 EC 8705 DSB 34 AVKS-7 CAT 3293 MACS 158 BNS-5 JS-335 JS 20-35 AGS-25 KBS-23 SL-955 PUNE-39 NRC-37 JS 9752 JS 71-05 JS 71-03 EC 85705 PUNE 30 MAUS 2 AVKS-4 EC 1720617 MACS 1488 AVKS 5 MACS 1460 PS 1618 JS 20-116 KDS 726 SL 958 RSC-1406 KHSB 2 AVKS 1 MACS 1410 KB 79 (46)	EC-241780 SL979 NRC 21 PUNE 32 AVKS 2 EC 546882 DS 3105 PS 1029 JS 93-05 MAUS 81 DSB 23-2 AVKS -6 PUNE-14 EC 8705 DSB 34 AVKS-7 CAT 3293 MACS 158 RKS-18 JS-335 MAUS-71 JS 20-35 AGS-25 KBS-23 SL-955 PUNE-39 NRC-37 JS 9752 MACS 450 JS 71-05 JS 71-03 EC 85705 PUNE 30 MAUS 2 AVKS-4 EC 1720617 MACS 1488 AVKS 5 MACS 1460 PS 1618 DURGA JS 20-116 KDS 726 SL 958 CAT 44 RSC-1406 KHSB 2 AVKS 1 MACS 1410 KB 79 104-31 JS 90-41 (52)	MAUS-71 EC-241780 SL979 NRC 21 PUNE 32 AVKS 2 EC 546882 DS 3105 PS 1029 JS 93-05 MAUS 81 DSB 23-2 AVKS -6 PUNE-14 EC 8705 DSB 34 AVKS-7 CAT 3293 MACS 158 RKS-18 BNS-5 JS-335 JS 20-35 AGS-25 KBS-23 SL-955 PUNE-39 NRC-37 JS 9752 MACS 450 JS 71-05 JS 71-03 EC 85705 PUNE 30 MAUS 2 AVKS-4 EC 1720617 MACS 1488 AVKS 5 MACS 1460 PS 1618 DURGA JS 20-116 KDS 726 SL 958 CAT 44 RSC-1406 KHSB 2 AVKS 1 MACS 1410 KB 79 104-31 JS 90-41 (53)	EC-241780 SL979 NRC 21 PUNE 32 AVKS 2 EC 546882 DS 3105 PS 1029 JS 93-05 MAUS 81 DSB 23-2 AVKS -6 PUNE-14 EC 8705 DSB 34 AVKS-7 CAT 3293 MACS 158 RKS-18 BNS-5 JS-335 MAUS-71 JS 20-35 AGS-25 KBS-23 SL-955 PUNE-39 NRC-37 JS 9752 MACS 450 JS 71-05 JS 71-03 EC 85705 PUNE 30 MAUS 2 AVKS-4 EC 1720617 MACS 1488 AVKS 5 MACS 1460 PS 1618 DURGA JS 20-116 KDS 726 SL 958 CAT 44 RSC-1406 KHSB 2 AVKS 1 MACS 1410 KB 79 KALITHUR 104-31 JS 90-41 (54)	PUNE-14 NRC 21 PUNE 32 AVKS 2 DS 3105 JS 93-05 MAUS 81 DSB 23-2 AVKS -6 DSB 34 AVKS-7 CAT 3293 RKS-18 BNS-5 JS 20-35 AGS-25 KBS-23 SL-955 PUNE-39 NRC-37 JS 9752 JS 71-05 PUNE 30 MAUS 2 104-31 EC 1720617 MACS 1488 AVKS 5 DURGA KDS 726 SL 958 CAT 44 RSC-1406 KHSB 2 AVKS 1 MACS 1410 KB 79 KALITHUR ACC No. 109 ACC No. 39 ACC No. 101 LB-5 (42)	PUNE-39 NRC 21 PUNE 32 AVKS 2 EC 546882 DS 3105 PS 1029 MAUS 81 DSB 23-2 AVKS -6 PUNE-14 DSB 34 CAT 3293 MACS 158 BNS-5 MAUS-71 JS 20-35 AGS-25 KBS-23 SL-955 NRC-37 JS 9752 MACS 450 JS 71-05 JS 71-03 PUNE 30 AVKS-4 MACS 1488 MACS 1460 DURGA SL 958 CAT 44 KHSB 2 AVKS 1 KB 79 ACC No. 37 KALITHUR ACC No. 101 (38)
Seed longevity Rank-4	104-31 KHSB 2 JS 90-41 CAT 44 DURGA CAT 3293 KDS 726 (7)	JS 90-41 CAT 44 DURGA CAT 3293 (4)	104-31 JS 90-41 CAT 44 DURGA KHSB 2 EC 1720617 MACS 450 JS 20-35 PUNE 32 (9)	104-31 JS 90-41 CAT 44 DURGA MACS 450 (5)	(0)	(0)	(0)	SL 979 MACS 158 MACS 450 PS 1029 EC 8705 EC-241780 JS 20-116 EC 85705 JS 90-41 EC 546882 MACS 1460 JS 71-03 (12)	JS 90-41 AVKS-7 JS 93-05 EC 85705 104-31 JS 20-116 AVKS 5 JS-335 KDS 726 MAUS 2 RSC-1406 SL979 (12)
Seed longevity Rank-5	(0)	104-31 KHSB 2 (2)	(0)	(0)	(0)	(0)	(0)	(0)	(0)

Values in parenthesis indicate the number of genotypes in each rank.

### Microsatellite marker diversity of genotypes

**Figure 5:**
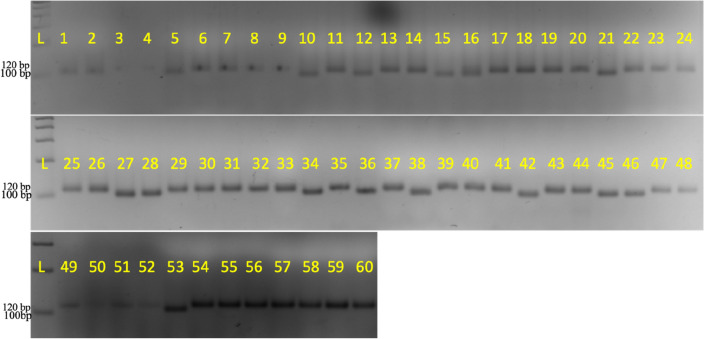
Banding pattern of BSOY 29 marker alleles in 60 genotypes (*L* Ladder) (The gel with serial number 49–60 was cropped from different gel and the full-length image is included in Supplementary Fig. S1).

**Figure 6:**
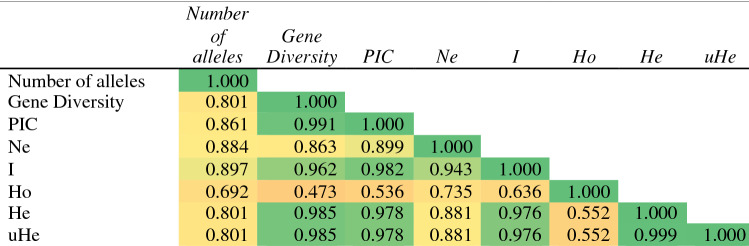
Correlation coefficient between different genetic diversity indices.

Genetic analysis of the sixty soybean genotypes was done using fifteen microsatellite markers. The presence and absence of alleles were scored as ‘1’ and ‘0’, respectively (Fig. 5, Supplementary Fig. S1). Totally, 38 alleles were observed from all the loci studied with a range of 1 to 5 and a mean of 2.533 per locus. The highest number of alleles per loci was observed for the marker BSOY 43 (5). The percentage of polymorphic loci among the genotypes was 93.33. Three alleles had allele frequency of less than 5% which are considered as rare. The mean frequency of minor and major alleles was 0.153 and 0.692, respectively (Table 5). Major allele frequency ranged from 0.350 to 1.000 and the minor allele frequency ranged from 0.000 to 0.417. BSOY 23 loci was monomorphic for the studied genotypes. The 60 genotypes were genetically diverse with a mean genetic diversity of 0.414. The genotypes were highly diverse for the BSOY 43 loci with a diversity of 0.769. The PIC value of the primers ranged from 0.000 to 0.735 with a mean of 0.359. BSOY 43 had the highest PIC value (0.735) as well as a greater number of alleles. A highly positive correlation of r = 0.861 (p < 0.00001) was found between number of alleles and PIC value (Fig. 6).

**Table 5:** Genetic diversity measures of the microsatellite markers in soybean genotypes.

Marker	Major allele frequency	Minor allele frequency (MAF)	Number of alleles	Gene diversity	PIC	No. of effective alleles	Shannon's information index	Observed heterozygosity	Expected heterozygosity	Unbiased expected heterozygosity	Number of genotypes with MAF
BSOY 18	0.817	0.183	2	0.299	0.255	1.427	0.476	0.000	0.299	0.302	11
BSOY 20	0.417	0.250	3	0.653	0.579	2.880	1.078	0.000	0.653	0.658	15
BSOY 28	0.700	0.300	2	0.420	0.332	1.724	0.611	0.000	0.420	0.424	18
BSOY 34	0.567	0.033	3	0.582	0.519	1.985	0.779	0.133	0.496	0.500	2
BSOY 36	0.800	0.033	3	0.331	0.294	1.495	0.591	0.000	0.331	0.334	2
BSOY 43	0.350	0.067	5	0.769	0.735	4.332	1.524	1.000	0.769	0.776	4
BSOY 47	0.700	0.017	3	0.468	0.427	1.832	0.800	0.017	0.454	0.458	1
BSOY 45	0.683	0.017	3	0.486	0.440	2.406	0.948	0.883	0.584	0.589	1
BSOY 1	0.600	0.150	3	0.555	0.491	2.247	0.938	0.000	0.555	0.560	9
BSOY 4	0.850	0.150	2	0.255	0.222	1.342	0.423	0.000	0.255	0.257	9
BSOY 6	0.617	0.383	2	0.473	0.361	1.897	0.666	0.000	0.473	0.477	23
BSOY 19	0.983	0.017	2	0.033	0.032	1.034	0.085	0.000	0.033	0.033	1
BSOY 23	1.000	0.000	1	0.000	0.000	1.000	0.000	0.000	0.000	0.000	–
BSOY 29	0.717	0.283	2	0.406	0.324	1.684	0.596	0.000	0.406	0.410	17
SATT 453	0.583	0.417	2	0.486	0.368	1.946	0.679	0.000	0.486	0.490	25
Mean	0.692	0.153	2.533	0.414	0.359	1.949	0.679	0.136	0.414	0.418

The average number of effective alleles, Shannon’s information index, observed heterozygosity, expected heterozygosity and unbiased heterozygosity of studied soybean genotypes were 1.949, 0.679, 0.136, 0.414, and 0.418 respectively (Table 5). Four out of 15 loci had multiple alleles in all the genotypes, with observed heterozygosity of 0.133, 1.00, 0.017 and 0.883, respectively for primers BSOY 34, BSOY 43, BSOY 47 and BSOY 45. The Shannon information index was higher for BSOY 43 marker with a value of 1.524 followed by BSOY 45 (0.948) predicting high diversity of the population and it is highly correlated with the genetic diversity of the population with a correlation coefficient of 0.962 (Fig. 6).

**Figure 7:**
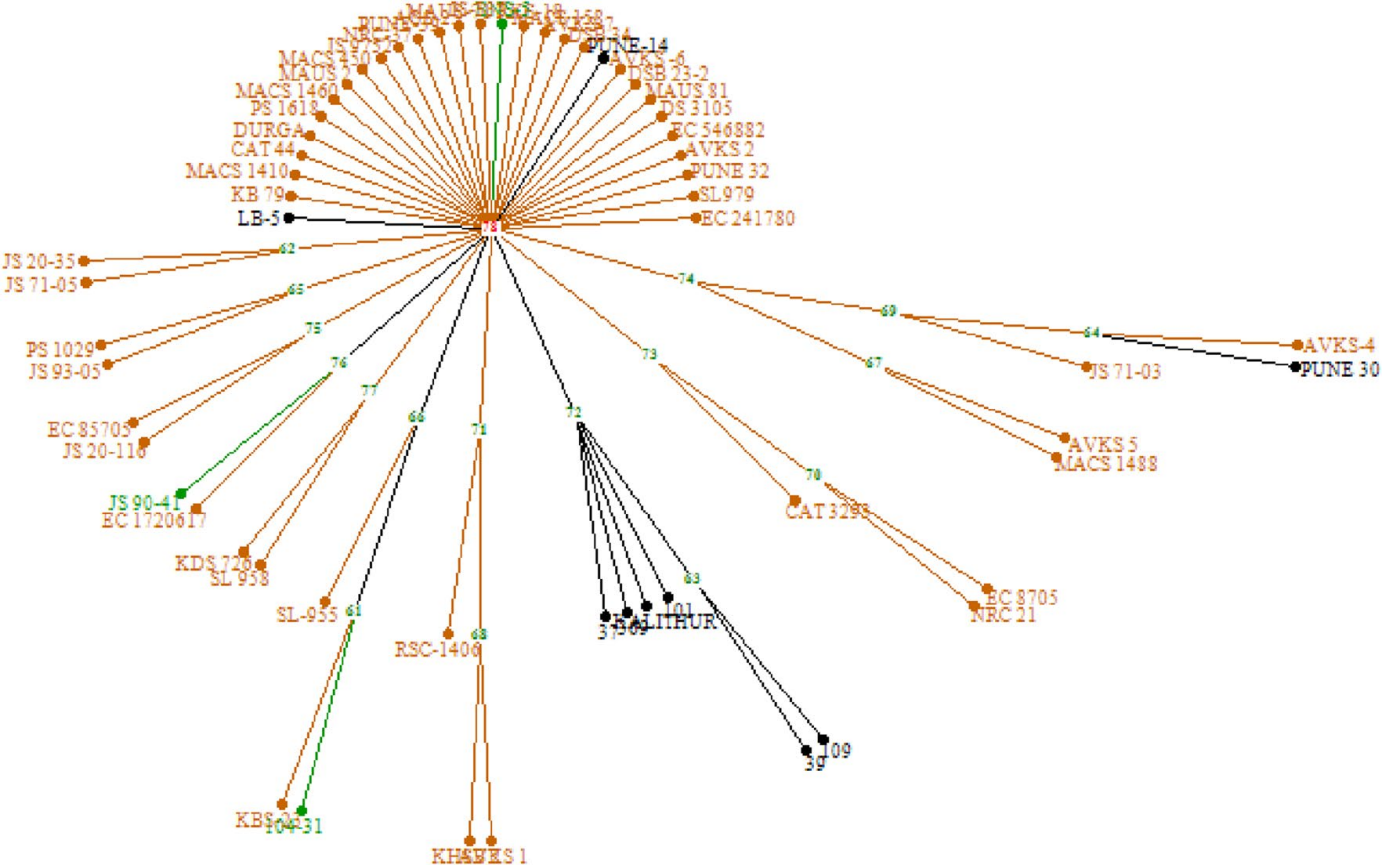
Hierarchical clustering of soybean genotypes based on UPGMA Euclidean distance (Black, Green, and Yellow colour lines indicate Black, Green, and Yellow seed coat colour genotypes).

The UPGMA based hierarchical clustering of the soybean genotypes based on the dissimilarity index grouped 30 genotypes into ten clusters with more than one genotype per cluster and the remaining 30 genotypes formed solitary clusters with only one genotype each (Fig. 7). One cluster with 6 genotypes solely consisted of black seed coat genotypes. Among the remaining three black seed coat colour genotypes, LB-5 and Pune 14 formed independent solitary clusters and Pune 30 was solitary in a sub-cluster. Three green seeded genotypes were grouped into different clusters, The yellow seed coat colour genotypes were also grouped in to different clusters and sub clusters.

## Discussion

The main purpose of this experiment in soybean is to identify the superior genotypes for seed longevity. Given the difficulty and time required for ageing under natural environmental conditions to assess the seed longevity of a genotype, accelerated ageing was proposed to test the longevity of a genotype or a segregant^37^. In soybean, an increase in seed yield combined with seed longevity in a single genotype is valuable for a breeder. Therefore, the identification of donors for seed longevity in soybean is more meaningful. Attempts have been made in the past to understand the genetic diversity for seed longevity in soybean^17,36,38^ and its influence on various seed characteristics^12,13,30,48^. Resources have also been allocated to understand the genetics of this complex polygenic trait^26,28,49^. However, the genetic resources identified for seed longevity for use in crop improvement programme are scanty.

This calls for screening genotypes for seed longevity and 60 genotypes representing different seed coat colour, seed size, and growth habit were evaluated for seed longevity using natural and accelerated ageing techniques. The germination percentage after ageing treatment has been frequently followed to estimate seed longevity^50,51^. The present study confirmed significant variability existing in tested soybean genotypes for seed longevity during seed dry storage. A similar observation was reported by Hosamani et al*.*^24^ in soybean, wherein, the viability of black genotypes had higher longevity than yellow seeded genotypes under natural and artificial ageing methods. Natural ageing for 3 years in three soybean species including cultivated and wild accessions exhibits significant variation for seed viability reduction between species, and wild accessions recorded higher seed longevity^17^.

Another objective of this study is a classification of diverse soybean genotypes with different seed coat colour and seed size based on their longevity which was tested after natural and accelerated ageing. In the PCA scatter diagram using seed germination values after both ageing, 11 genotypes with higher seed longevity were found in the first and fourth quadrants with higher loadings. Among them, seven were black, three with yellow, and one was green (BNS-5) seed coat colour genotypes. The PCA scatter plot has been used to identify stress tolerant, high productivity genotypes in different crops^52,53^. The genotypes were also grouped based on molecular markers. The molecular marker system has become an essential part of the genetic diversity analysis and for crop improvement programme^54^. The discriminatory power of a marker is well explained by PIC value by using the number of alleles and their frequencies^55^. Among the studied microsatellite markers, BSOY 43 was highly polymorphic having higher number of alleles and PIC value. PIC values above 0.5 are said to be consistent with the usefulness of the marker^56^. The informativeness of the markers on genetic diversity was being estimated using PIC value by many researchers^55,57,58^ and made a positive correlation with number of alleles. The high percentage of polymorphic loci among the genotypes suggests that the genotypes used for the study were diverse^57^. Further, the rare alleles with the occurrence of less than 5% allele frequency are also observed, hence, the genotypes used in the study represent both recently cultivated genotypes as well as rare genotypes^55^. A positive correlation between genetic diversity, PIC, and Shannon’s information index was also observed in this study. The Shannon information index is a measure of the degree of uncertainty in predicting the species of a sample, which is related to the diversity of a population^59^.

The molecular marker clustering also supports the results observed in PCA that the genotypes used for the present study are highly diverse at the genome level also. Like in PCA, one of the clusters formed using molecular markers contain majority of black seed coat colour genotypes. The clustering of genotypes using genotypic and phenotypic data are largely in agreement. The observations are in line with the finding made by Jeong et al.^60^ and Kachare et al.^57^ wherein, the clusters obtained using phenotypic diversity was in accordance with the cluster found after performing SSR marker diversity.

The major objective of the present study is to identify the genotypes with higher seed longevity. After different duration of ageing of seeds under natural conditions and accelerated ageing, the seeds were tested for viability using germination percent. A significant positive correlation was found between natural ageing after 8th month till 20th month of storage under ambient conditions and the accelerated ageing method. Hosamani et al.^24^ and Matera et al.^38^ also studied the association between natural and accelerated ageing in soybean and reported highly significant correlation between them in detecting seed longevity. The method has been widely used in crops like rice^61^, wheat^62^, chickpea^33^, Brassica^63^, soybean^26,28,36^, and palm^64^ for seed longevity. Accelerated ageing method is based on the principle of seed survival curve, that explains the relationship between seed viability and storage period. Accelerated ageing reduces the lag phase of a seed in declining its viability during storage^37^ by subjecting the seed to conditions that hasten the deterioration process. Such artificial ageing method is reliable, time saving, and useful for the quick assessment of segregants or genotypes in breeding. In the present study we have adapted both natural and accelerated ageing techniques to identify genotypes with higher seed longevity.

Based on germination after natural and accelerated ageing methods, the genotypes were classified into different longevity rank groups. Majority of the genotypes were found in low seed longevity rank group of SR 3 and SR 4. Only a few genotypes were consistently found in the highest seed longevity group of SR 1. The two genotypes, i.e., ACC No. 369 and ACC No. 39 were frequently found in SR 1 group in both ageing methods and hence, the highest seed longevity among the genotypes. Both of them were found to be black seed coat colour genotypes. The black seed coat soybean genotypes were found to have relatively higher longevity^13,28,65^. In addition to these two black seed coat genotypes, other black seed genotypes, ACC No. 37, ACC No. 109, and LB-5 were found either in SR 1 or in SR 2 group. Seed longevity was also found to be associated with seed coat colour. The seed coat colour association with seed longevity observed in this study is in harmony with the earlier findings^13,17,24,28,65^. Kuchlan et al.^13^ reported that, black seeded genotypes have a minor gap between the seed coat and cotyledon, lesser pores on the surface of the seed coat, and greater lignin content in the seed coat, which makes them less susceptible to mechanical damage and deteriorative changes during ageing, than other seed coat colours. All the black seeded genotypes with high seed longevity had small seed size and lower 100 seed weight. The 100 seed weight ranged from 9.95 to 13.07 g. The other two black genotypes, Pune 14 and Pune 30 which did not show higher longevity had bigger seed size (15.20 and 17.10, respectively). It can be concluded that along with black seed coat colour, seed size are the determining factors for seed longevity in soybean^30^.

Finally, in the study, contrasting accessions have been identified, which can be used for additional experiments to determine QTLs controlling the trait and also for breeding commercial lines. For this objective, the contrasting, yellow genotype with low longevity and black genotype with higher longevity have been crossed, and the true F_1_ plants have been identified using molecular and morphological markers and the F_2_ generation is being grown to develop RILs suitable for mapping seed longevity-related traits.

## Supplementary Information

Supplementary Information.

## Data Availability

The datasets generated during and/or analysed during the current study are available from the BioStudies repository of EMBL-EBI. The Accession Number is S-BSST814. The below link can be used to access the data https://www.ebi.ac.uk/biostudies/studies/S-BSST814?key=e287dfc1-07ae-4491-b44f-25d6f75cf80c.
