# Supplementary material for: Evaluation of diverse soybean genotypes for seed longevity and its association with seed coat colour
Source: Sci Rep. 2023 Mar 15;13:4313. doi: 10.1038/s41598-023-31071-3 (PMC10017797; doi:10.1038/s41598-023-31071-3)
Supplement: Supplementary file 1 — Supplementary Information. [file 41598_2023_31071_MOESM1_ESM.docx]

**Evaluation of diverse soybean genotypes for seed longevity and its association with seed coat colour**

NAFLATH T. V.^1^, RAJENDRAPRASAD S.^1^ AND RAVIKUMAR R. L.^2*^

1. Department of Seed Science and Technology, College of Agriculture, UAS, GKVK, Bangalore, Karnataka, India-560 065
2. Department of Plant Biotechnology, College of Agriculture, UAS, GKVK, Bangalore, Karnataka, India - 560 065

*Corresponding author: [rlravikumar@rediffmail.com](mailto:rlravikumar@rediffmail.com)

T. V. NAFLATH- ORCID: ID: <https://orcid.org/0000-0002-1193-033X>

| Genotype number | Genotypes | Seed coat colour | 100 seed weight (g) | Genotype number | Genotypes | Seed coat colour | 100 seed weight (g) |
| --- | --- | --- | --- | --- | --- | --- | --- |
| 1 | EC 241780 | Yellow | 13.20 | 31 | MACS 450 | Yellow | 16.27 |
| 2 | SL979 | Yellow | 19.65 | 32 | JS 71-05 | Yellow | 14.75 |
| 3 | NRC 21 | Yellow | 14.32 | 33 | JS 71-03 | Yellow | 16.29 |
| 4 | PUNE 32 | Yellow | 12.56 | 34 | EC 85705 | Yellow | 17.73 |
| 5 | AVKS 2 | Yellow | 13.77 | 35 | RSC-1406 | Yellow | 14.21 |
| 6 | EC 546882 | Yellow | 15.23 | 36 | MAUS 2 | Yellow | 18.05 |
| 7 | DS 3105 | Yellow | 16.07 | 37 | AVKS-4 | Yellow | 14.58 |
| 8 | PS 1029 | Yellow | 18.14 | 38 | MACS 1410 | Yellow | 13.89 |
| 9 | JS 93-05 | Yellow | 15.00 | 39 | EC 1720617 | Yellow | 15.57 |
| 10 | MAUS 81 | Yellow | 16.32 | 40 | MACS 1488 | Yellow | 15.79 |
| 11 | DSB 23-2 | Yellow | 15.27 | 41 | AVKS 5 | Yellow | 14.98 |
| 12 | AVKS -6 | Yellow | 13.68 | 42 | MACS 1460 | Yellow | 15.75 |
| 13 | KHSB 2 | Yellow | 14.84 | 43 | PS 1618 | Yellow | 14.23 |
| 14 | EC 8705 | Yellow | 15.19 | 44 | DURGA | Yellow | 14.69 |
| 15 | DSB 34 | Yellow | 15.97 | 45 | JS 20-116 | Yellow | 15.13 |
| 16 | AVKS-7 | Yellow | 15.68 | 46 | KDS 726 | Yellow | 17.45 |
| 17 | CAT 3293 | Yellow | 13.45 | 47 | SL 958 | Yellow | 15.23 |
| 18 | MACS 158 | Yellow | 15.65 | 48 | CAT 44 | Yellow | 18.83 |
| 19 | RKS-18 | Yellow | 16.40 | 49 | 104-31 | Green | 14.07 |
| 20 | AVKS 1 | Yellow | 15.03 | 50 | BNS-5 | Green | 10.02 |
| 21 | JS-335 | Yellow | 15.63 | 51 | JS 90-41 | Green | 10.78 |
| 22 | MAUS-71 | Yellow | 15.40 | 52 | PUNE 30 | Black | 17.10 |
| 23 | JS 20-35 | Yellow | 16.55 | 53 | PUNE-14 | Black | 15.20 |
| 24 | AGS-25 | Yellow | 11.37 | 54 | ACC. No. 37 | Black | 13.07 |
| 25 | KBS-23 | Yellow | 16.23 | 55 | ACC. No. 369 | Black | 11.52 |
| 26 | SL-955 | Yellow | 16.99 | 56 | KALITHUR | Black | 12.07 |
| 27 | PUNE-39 | Yellow | 13.97 | 57 | ACC. No. 39 | Black | 10.66 |
| 28 | NRC-37 | Yellow | 14.32 | 58 | ACC. No. 109 | Black | 10.66 |
| 29 | JS 9752 | Yellow | 12.43 | 59 | ACC. No. 101 | Black | 10.06 |
| 30 | KB 79 | Yellow | 15.15 | 60 | LB-5 | Black | 9.95 |

**Supplementary table S1. List of soybean genotypes used for the study, their seed coat colour and seed size**

| Primer number | Primer name | Motif | Forward sequence | Reverse sequence | Annealing temperature (^0^C) |
| --- | --- | --- | --- | --- | --- |
| **BSOY18** | BARCSOYSSR_01_0211 | (AG)16 | CCTTCTATCCGCAAAACCCT | ATATCACTCCCTCCCCAACC | 55.5 |
| **BSOY20** | BARCSOYSSR_01_1484 | (TC)18 | CTTCTCTCAGCACCCTCCAC | AACCCTTCTTCCACTTCCGT | 54.5 |
| **BSOY28** | BARCSOYSSR_04_0414 | (CT)17 | CCATTCTACAATCATGCCCC | AGAAGCTGGCTAAGATGGCA | 57.5 |
| **BSOY34** | BARCSOYSSR_08_1190 | (TC)16 | AATCATCTCCCAAGGAGTGC | TGGCAAACTTTGGTCATTCA | 55.0 |
| **BSOY36** | BARCSOYSSR_08_1346 | (GT)16 | TATGTGTGTGTGTGTGGGGG | GTGCTGGCTTTTCAACCAAA | 57.5 |
| **BSOY43** | BARCSOYSSR_09_0179 | (AG)20 | ACCAATTGCCAACAACACAA | GTCCTACAAGGCCCACAAAA | 55.0 |
| **BSOY47** | BARCSOYSSR_10_0267 | (CT)17 | ACATCGTTCTCCCTCTCCCT | CCTTCTTCCCAGAACCATCA | 57.5 |
| **BSOY45** | BARCSOYSSR_11_1391 | (TA)20 | CTTTTGGCACCACAAATTCC | TGGGCTCATTACGAGCATCT | 51.5 |
| **BSOY1** | BARCSOYSSR_12_0930 | (GA)18 | CCACCACTCATCAACACCTG | CGTCAAGGTTCCCTTACCCT | 54.5 |
| **BSOY4** | BARCSOYSSR_14_1271 | (CT)16 | AAGGAAGGAAAACCCATGCT | GGGACCACAGCGTTGAATTA | 51.5 |
| **BSOY6** | BARCSOYSSR_15_1563 | (TC)18 | GCGCAAGCACTGAATGTCA | GCGTCACTAACACCTATAACA | 51.5 |
| **BSOY19** | BARCSOYSSR_18_1942 | (TC)19 | CTCCTCATGCTTGGCAAAAT | ATGAGAACGCTGAAAAGGGA | 55.0 |
| **BSOY23** | BARCSOYSSR_19_0884 | (AG)16 | CCAAGTAGCGGGGTTACAAA | GACCATAGTAGCGAGGGCTG | 54.5 |
| **BSOY29** | BARCSOYSSR_19_1324 | (TC)16 | TCTCTTTTCACGGTGGCTTC | AAGGAGTGGTTGTGGGTTTG | 57.5 |
| **SATT453** | BARCSOYSSR_11_1468 | (ATT)14 | GCGGAAAAAAAACAATAAACAACA | TAGTGGGGAAGGGAAGTTACC | 55.5 |

**Supplementary table S2. List of SSR primers, their sequence with annealing temperature**


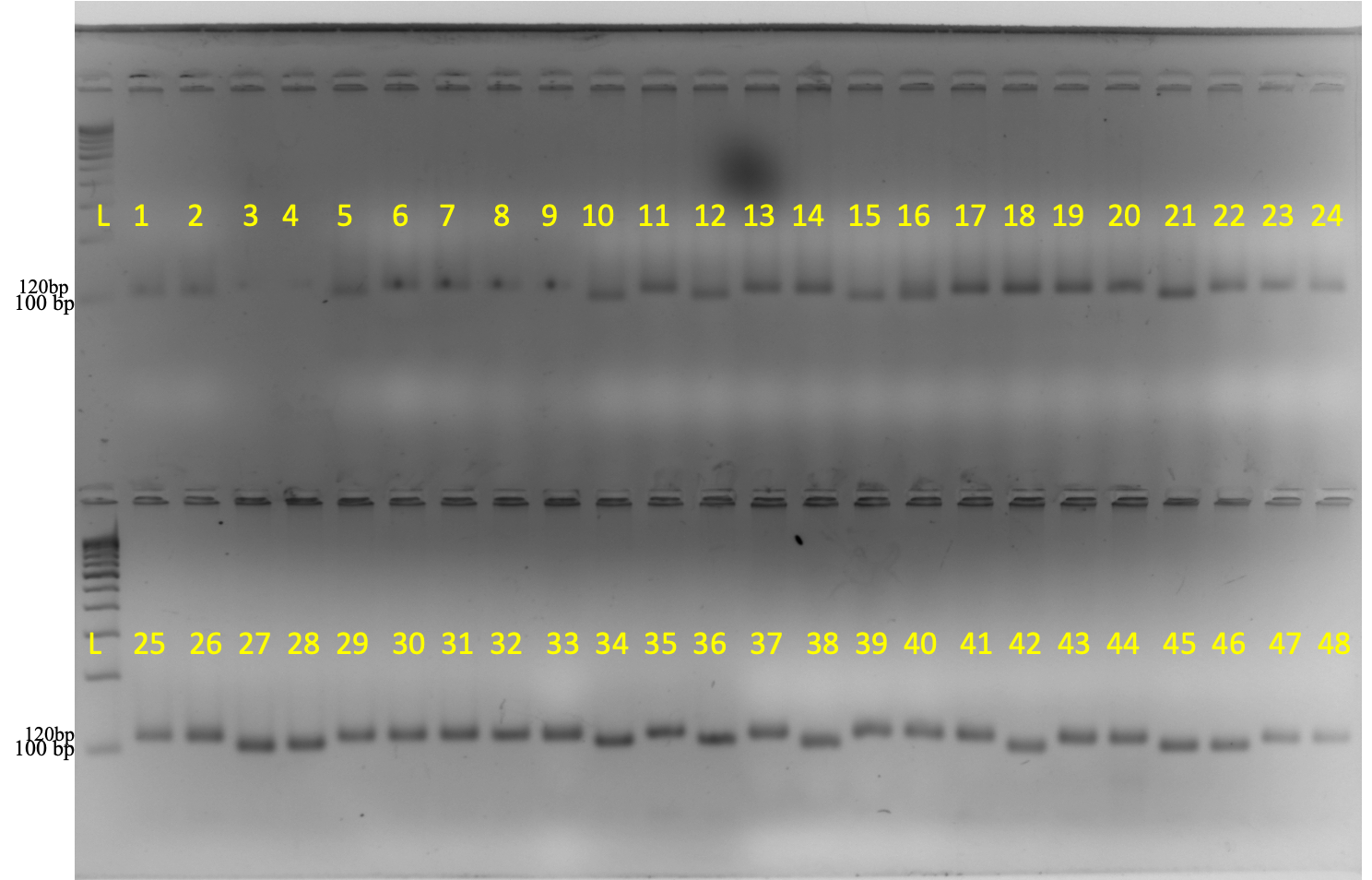


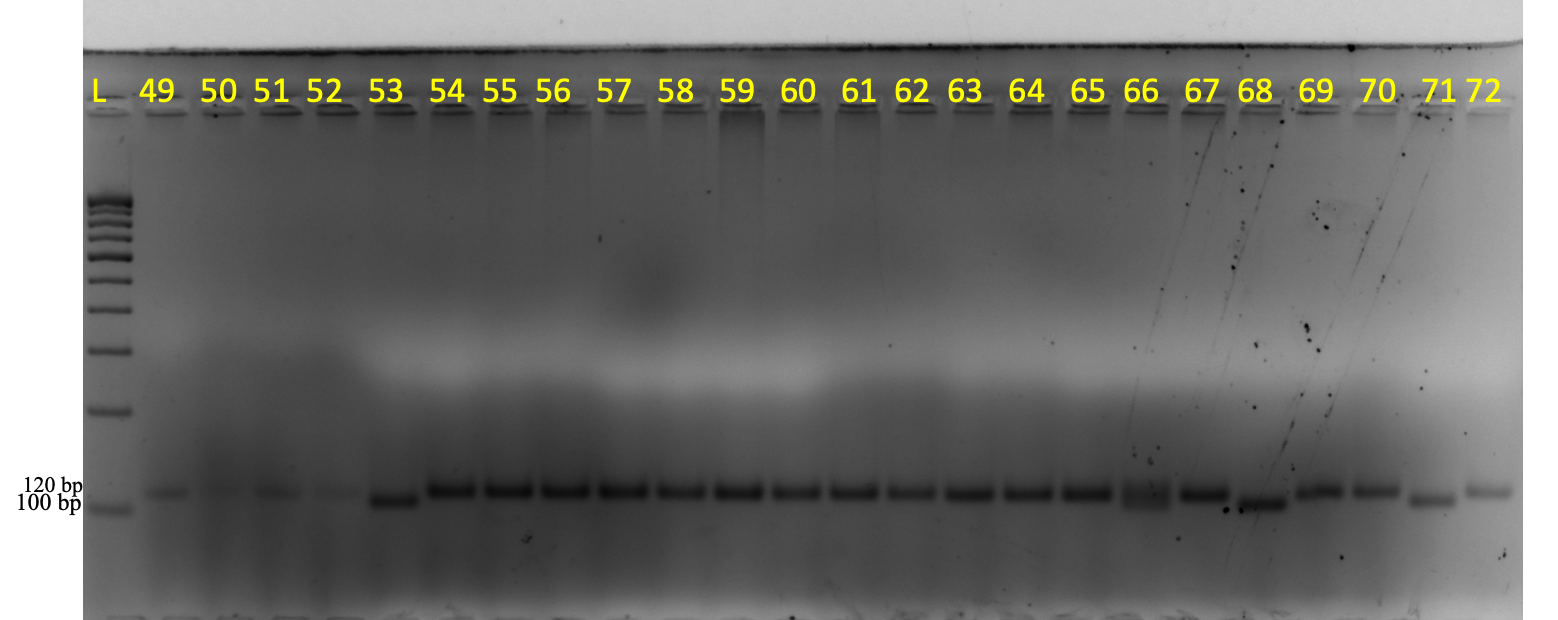


**Supplementary fig. 1. Full-length image of the cropped gel picture (Fig. 5) of BSOY 29 banding pattern**
